# Comparative Phosphoproteomics Reveals an Important Role of MKK2 in Banana (*Musa spp*.) Cold Signal Network

**DOI:** 10.1038/srep40852

**Published:** 2017-01-20

**Authors:** Jie Gao, Sheng Zhang, Wei-Di He, Xiu-Hong Shao, Chun-Yu Li, Yue-Rong Wei, Gui-Ming Deng, Rui-Bin Kuang, Chun-Hua Hu, Gan-Jun Yi, Qiao-Song Yang

**Affiliations:** 1 State Key Laboratory for Conservation and Utilization of Subtropical Agro- bioresources, South China Agricultural University, Guangzhou, 510640, China; 2 Institute of Fruit Tree Research, Guangdong Academy of Agricultural Sciences, Guangzhou, 510640, China; 3 Key Laboratory of South Subtropical Fruit Biology and Genetic Resource Utilization, Ministry of Agriculture, Guangzhou, 510640, China; 4 The Guangzhou Research Branch of the National Banana Improvement Center, Guangzhou, 510640, China; 5 Institute of Biotechnology, Cornell University, Ithaca, NY, USA; 6 Key Laboratory of Horticultural Plant Biology of the Ministry of Education, College of Horticulture and Forestry Sciences, Huazhong Agricultural University, Wuhan, China

## Abstract

Low temperature is one of the key environmental stresses, which greatly affects global banana production. However, little is known about the global phosphoproteomes in *Musa spp*. and their regulatory roles in response to cold stress. In this study, we conducted a comparative phosphoproteomic profiling of cold-sensitive Cavendish Banana and relatively cold tolerant Dajiao under cold stress. Phosphopeptide abundances of five phosphoproteins involved in MKK2 interaction network, including MKK2, HY5, CaSR, STN7 and kinesin-like protein, show a remarkable difference between Cavendish Banana and Dajiao in response to cold stress. Western blotting of MKK2 protein and its T31 phosphorylated peptide verified the phosphoproteomic results of increased T31 phosphopeptide abundance with decreased MKK2 abundance in Daojiao for a time course of cold stress. Meanwhile increased expression of MKK2 with no detectable T31 phosphorylation was found in Cavendish Banana. These results suggest that the MKK2 pathway in Dajiao, along with other cold-specific phosphoproteins, appears to be associated with the molecular mechanisms of high tolerance to cold stress in Dajiao. The results also provide new evidence that the signaling pathway of cellular MKK2 phosphorylation plays an important role in abiotic stress tolerance that likely serves as a universal plant cold tolerance mechanism.

During growth and development plants are subjected to numerous biotic and abiotic stresses. Low temperature is one of the most important and common plant abiotic stress factors. Low temperature has been reported to shift the thermodynamic equilibrium when there is an increased likelihood that non-polar side chains of proteins become exposed to the aqueous medium of the cell, which can directly affect the stability and the solubility of many globular proteins^1^. This leads to a disturbance in the stability of proteins or protein complexes, and, therefore, to a disruption of metabolic regulations. Confronted with these disruption, some plants have the ability to adapt through cellular regulatory mechanisms based on protein synthesis^2^, membrane composition changes^3^,^4^, and activation of active oxygen scavenging systems^5^. These adaptive mechanisms rely in part on gene induction^6^ and protein modifications^7^.

*Musa spp*., which originated in tropics are giant perennial herbaceous monocots. They are vital staple food in many African countries and the most popular fruits in industrialized countries^8^. Generally, plants from most temperate regions have the capacity to develop increased freezing tolerance after being exposed to low, nonfreezing temperatures, which is called cold acclimate^9^. Many important crops originated in the tropics and subtropics, such as rice, maize, soybean, cotton, and tomato, lack the cold acclimation mechanism and are sensitive to chilling stress^10^, so do *Musa spp*. Although *Musa spp*. appear to lack a cold acclimation mechanism, they exhibit a high degree of genetic variability for cold tolerance, in which Cavendish Banana (*Musa spp*. Cavendish; AAA Group) is more cold sensitive than Dajiao (*Musa spp*. Dajiao; ABB Group). When the temperature drops to 8 °C, Cavendish Banana growth is arrested, injury is inflicted^11^, and irreversible damage often occurs with temperatures below 12 °C^12^. Dajiao however, has superior cold tolerance, enabling to tolerate temperatures of 0–4 °C^13^. It has been proposed as a potential germplasm resource of cold tolerance in banana breeding^6^.

Reversible modification on specific residues of plant proteins, e.g. phosphorylation, is a common signaling event that occurs in regulating protein function in response to both abiotic and biotic stresses. Dynamic changes of the phosphoproteome in response to short-term (5 min or less) exposure to 4 °C in radioactive pulse-labelling of *Arabidopsis* cell suspension cultures and similar experiment on rice roots were identified by 2-dimensional gel electrophoresis (2DGE) analysis^14^,^15^. The results of these two studies reveal that during cold exposure some proteins are phosphorylated by protein kinases, whereas others are dephosphorylated by protein phosphatases. Presently, many different kinases or phosphatases under cold stress had been identified, such as Calcium dependent protein kinases (CDPK)^16^, Ca^2+^/calmodulin-dependent protein kinases (CAMK)^17^, MAPK (mitogen activated protein kinase) module^7^,^18^, STY kinases^19^, CBL-interacting protein kinase (CIPK)^20^,^21^, OST1^22^ and AP2C1 proteins^23^. Phosphorylation changes of MAPK cascades in plant cold resistance have been well reported, particularly in model plants. For example, the MKK2 pathway was found to mediate cold and salt stress signaling in *Arabidopsis*^18^, phosphorylation of *Arabidopsis thaliana* MEKK1 via Ca^2+^ signaling is a part of the cold stress response^24^ and membrane rigidification functions upstream of the MEKK1- MKK2-MPK4 cascade during cold acclimation in *Arabidopsis thaliana*^7^. However, little information has been reported in non-model plants, particularly in *Musa spp*., which sparked a profound interest to us regarding the potential role of this type of regulation and its mechanisms. In addition, the relationship and interactions among the global cold-responsive phosphoproteins and MAPK cascades have not been well characterized in plant. In recent years, mass spectrometry (MS)-based phosphoproteomic analysis has provided a more effective, sensitive and accurate method allowing for global phosphorylation study in plants subjected to variable and different stresses^25^.

Quantitative proteomic analyses in our group reveal that molecular mechanisms leading to the higher cold resistance in Dajiao are closely associated with the anti- oxidation capacity, and cell wall stabilization^11^. Recently we conducted a comparative transcriptomics analysis of Cavendish Banana and Dajiao in response to cold stress and showed that the rapid activation and selective induction of ICE1 and MYBS3 cold tolerance pathways in Dajiao, along with expression of other cold-specific genes, appear to be one of the main mechanisms for the higher cold resistance in Dajiao than Cavendish Banana^6^. However, the mechanisms of cold acclimation involving the possible regulation by phosphoproteins and their signaling networks in both Dajiao and Cavendish Banana under cold stress has not been reported and remains unknown.

In this study, a MS-based comparative phosphoproteomic approach was used to identify and characterize global phosphorylated proteins under cold stress in both Dajiao, which was collected from a subtropical region of China, and Cavendish Banana, a cold-sensitive species utilized as a control. We used a liquid chromatography-tandem mass spectrometry (LC-MS/MS) analysis of TMT 6-plex labeled samples that were subjected to consecutive enrichment by Fe^3+^-IMAC and titanium dioxide (TiO_2_) affinity chromatography. Changes in abundance of proteins and their phosphorylated counterparts were compared between Dajiao and Cavendish Banana after cold-stress conditions. In addition, to identify the key phosphoproteins and their signal networks responsible for cold-tolerance in Dajiao, protein-protein interactions among the differentially expressed phosphoproteins were analyzed and mapped using STRING v. 10. Moreover, the cold stress tolerance mechanism presumptively regulated by phosphorylation signaling network is discussed.

## Results

### Physiological Responses of Cavendish Banana and Dajiao to Cold Stress

**Figure 1:**
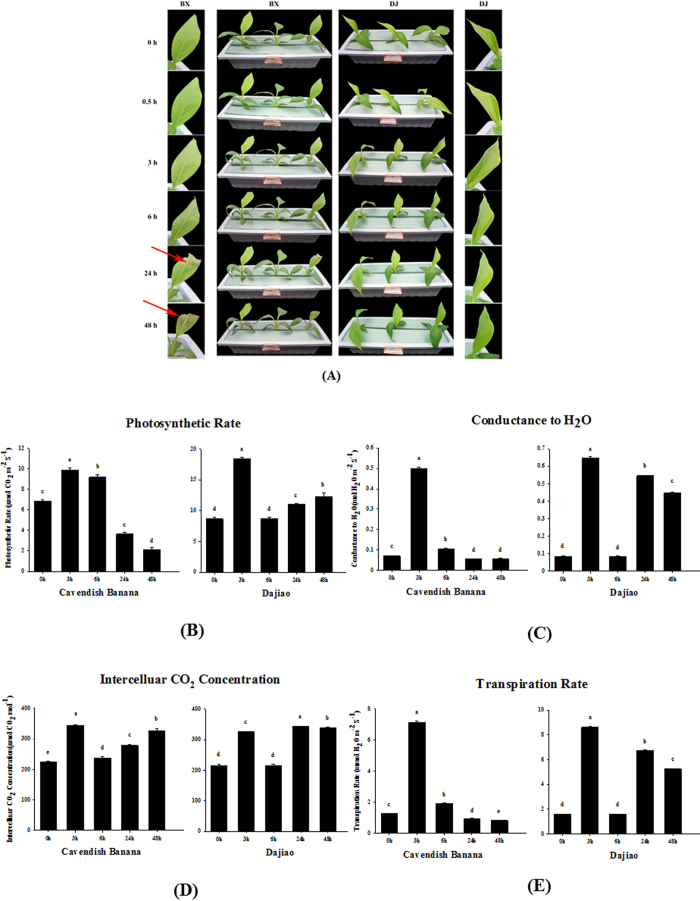
Physiological responses of Cavendish Banana and Dajiao under cold stress. Five leaf stage seedlings were treated at 10 °C for 0, 0.5, 3, 6, 24 and 48 h (**A**). The photosynthetic rate (Photo), conductance to H_2_O (Cond), the intercellular CO_2_ concentration (Ci) and transpiration rates (Trmmd) are shown in (**B**–**E**), respectively. Each column means ± S.D. of three biological replicates with each having three to five technical replicate measurements. The different lowercase letters labeled above columns indicate a significant difference at p ≤ 0.05 between the columns by Duncan’s test using SPSS statistical software (version 16.0, SPSS Inc. Chicago, IL). The columns with the same letters mean no significant difference (p > 0.05) between each other.

A significant phenotypic difference of Cavendish Banana and Dajiao seedlings under cold stress was that the Cavendish Banana leaves drooped after 6 h of cold treatment and the second and third leaves from the top displayed severe necrosis and wilting symptoms after 48 h of cold treatment at 10 °C, while Dajiao leaves remained resistant (Fig. 1A)^6^. Thus, cold treatment at 10 °C was used for this study.

To understand the different changes that occured during cold stress in Cavendish Banana and Dajiao seedlings, photosynthetic characteristics were measured and used to select the most appropriate time points for subsequent phosphoproteomic analysis. After 10 °C cold stress, the photosynthetic rate (Photo), conductance to H_2_O (Cond), intercellular CO_2_ concentration (Ci) and transpiration rate (Trmmd) of Dajiao showed similar changes during the time course. All of them increased significantly at 3 h cold stress, backed to the normal level at 6 h and then gradually increased again after 24 h and 48 h (Fig. 1). For Cavendish Banana, the change of Ci showed a similar trend with Dajiao, the Photo, Cond and Trmmd increased significantly at 3 h, then backed to near normal level at 6 h, but still remained normal level at 24 h and 48 h (Fig. 1). These results revealed that low temperature photo-inhibition^26^ occurred in both Cavendish Banana and Dajiao, but apparently Dajiao had a stronger ability to recover from cold stress than Cavendish Banana.

According to these data, 6 h is the observed decay time point for cellular proteins to play an adaptation role in cold resistance. Given the fact that 3 h data point appears to be the apex point of photosynthetic response plus protein modifications often occur at an early stage of protein translation, we chose 3 h as the time point for phosphoproteomic analysis, with 0 h being used as a control.

### Identification of Phosphosites in Cavendish Banana and Dajiao Under Cold Stress

The results from phosphoproteomic analysis on Cavendish Banana and Dajiao leaves after 3 h cold stress compared to no stress control are summarized in Table 1. A total of 679 unique phosphopeptides containing 772 individual phosphosites from 529 individual phosphoproteins were confidently identified from Cavendish Banana, in which 438 phosphopeptides and 483 individual phosphosites belonging to 352 phosphoproteins were quantified. In Dajiao, a total of 241 phosphopeptides and 271 individual phosphosites from 207 phosphoproteins were identified, in which 167 phosphopeptides containing 188 individual phosphosites from 144 phosphoproteins were quantified (Table 1, see Supplementary Table S1).

Of the 772 distinct phosphosites found in Cavendish Banana, 653 (84.6%) were phosphorylated at serine (pSer), 116 (15.0%) at threonine (pThr), and 3 (0.4%) at tyrosine (pTyr) residues. This result is in good agreement with previous report in other plants: 89.3% pSer, 10.2% pThr, and 0.5% pTyr in soybean^27^ and 89.4% pSer, 9.5% pThr, and 1.1% pTyr in cotton^28^. However, the result of Dajiao showed a considerable difference. Of the 271 distinct phosphosites, 209 (77.1%), 57 (21.0%) and 5 (1.9%) were pSer, pThr and pTyr respectively (Table 1, see Supplementary Table S1).

Of the 679 unique phosphopeptides identified in Cavendish Banana, 586 (86.3%), 88 (13.0%) and 5 (0.7%) were found to be singly, doubly, and multiply phosphorylated peptides, respectively. The same distribution of 213 (88.4%), 27 (11.2%) and 1 (0.4%), respectively, occurred in Dajiao (Table 1, see Supplementary Table S1). These values are very different from those reported for cotton (33.4%, 48.3%, and 18.3%)^28^, but are quite consistent with those found in *Arabidopsis* (80.9%, 19.1%, and 0%)^29^. This difference is conceivable with the use of different methodologies or biological systems, where different tissue and organism under a particular condition may have a special phosphoproteome profile.

In order to analyze conservation of all identified phosphosites, we compared phosphorylation patterns of orthologous phosphosites among Cavendish Banana, Dajiao and eight common plant species (*Oryza sativa japonica* (Japanese rice); *Brachypodium distachyon*; *Sorghum bicolor* (sorghum); *Zea mays* (maize); *Arabidopsis thaliana* (thale cress); *Glycine max* (soybean); *Solanum lycopersicum* (tomato); *Vitis vinifera* (wine grape)) from existing UniprotKB database. The results show that in Cavendish Banana and Dajiao the phosphorylation sites on Ser, Thr and Tyr residues yield relatively higher evolutionary conservation (at average of 20.5%) than unphosphosites on the three residues (at average of 18.0%) across different plant species (see Supplementary Table S2).

### Phosphoproteome Characterization, Classification and Phosphopeptide Motif Discovery

Blast2GO was used to annotate and classify phosphoproteins by biological process (BP), cellular component (CC) and molecular function (MF) categories at a GO annotation level 2. In Dajiao dataset, number of phosphoproteins related to BP, CC, and MF was 179, 85, and 189, respectively (see Supplementary Fig. S2A and Table S3). In Cavendish Banana dataset, information related to BP, CC, and MF was obtained for 468, 175, and 476 phosphoproteins, respectively (see Supplementary Fig. S2B and Table S3). Most of the annotated phosphoproteins of Cavendish Banana and Dajiao are involved in binding, cellular process and catalytic activity, consistent with the findings of previous investigation^28^,^30^.

**Figure 2:**
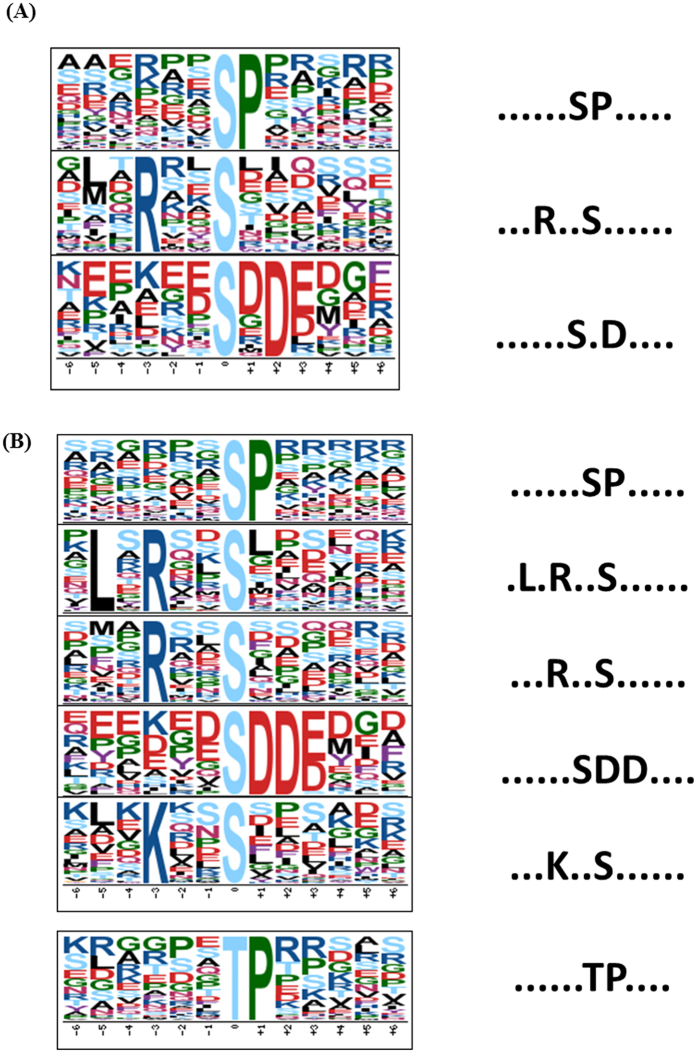
Motif analysis of all the identified phosphosites. (**A**) Significantly enriched phosphorylation motif of Dajiao under cold stress. (**B**) Significantly enriched phosphorylation motif of Cavendish Banana under cold stress.

Motif analysis can be used for evaluating sequence conservation at phosphosites and for predicting the associated kinases. Motif-X analysis shows that three major phosphorylation motifs are enriched in Dajiao and six motifs were found in Cavendish Banana (Fig. 2A,B), while three of them ([SP], [RXXS], [SXD]) were found in both Cavendish Banana and Dajiao. Those phosphorylation motifs were used to search against the relevant databases to find the specific protein kinases^30^,^31^,^32^,^33^,^34^,^35^ them may be associated with. [SP] and [TP] motifs are the typical proline-directed motifs, which are potential substrates of mitogen-activated protein kinase (MAPK), cyclin-dependent kinase, and cyclin- dependent kinase-like. [RXXS] motif, recognized by CaMK II is another well-known motif. [SXD] (including [SDD]) motif is all acidic motifs and were recognized by casein kinase-II(CK II) that is involved in cell cycle control, DNA repair, circadian rhythm regulation, and other metabolic pathways.

### Statistical Analysis of Quantified Phosphopeptides in Biological Replicates

To assess the quantitative precision and reproducibility, the variance of the three biological replicates of data in both Dajiao and Cavendish Banana sets were calculated to determine the threshold of significant ratio changes. According to the calculation method of Lan P. *et al*. and Gan CS *et al*.^36^,^37^, the largest internal errors of Dajiao sets were 0.44, corresponding to a 1.35-fold change, which was used as a threshold of significant changes in response to cold stress. By using the same method, the cutoff for significant ratio changes of Cavendish Banana under cold stress is ±1.34-fold change (see Supplementary Fig. S3). All the differentially expressed proteins identified were listed in Supplementary Table S1.

**Figure 3:**
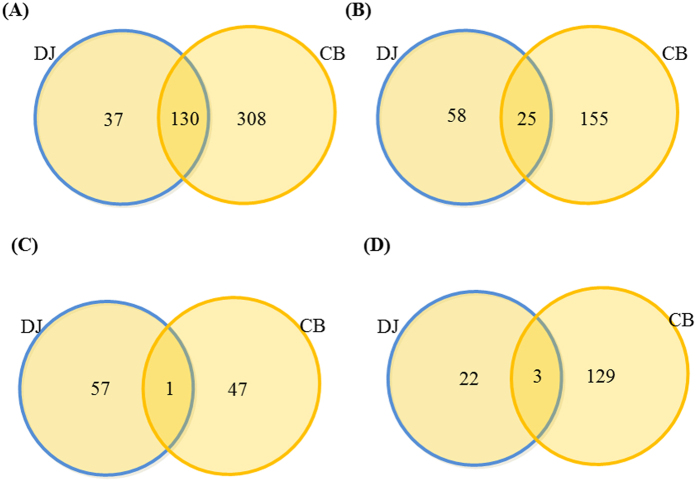
Venn diagram of number of phosphopeptides identified in Dajiao and Cavendish Banana leave phosphoproteome. (**A**) The number of quantified phosphopeptides; (**B**) the number of differentially expressed phosphopeptides; (**C**) the number of phosphopeptides with increased abundance; (**D**) the number of phosphopeptides with decreased abundance in Dajiao (DJ) and Cavendish Banana (CB).

As shown in Table 1, more than twice number of phosphopepitdes were identified and quantified in Cavendish Banana than in Dajiao. Surprisingly, only small percentage of significantly changed phosphopeptides was commonly found in both species (Fig. 3B). Interestingly, in both datasets we found large portion (~40–50%) of phosphopeptides, which abundance significantly changed in response to 3 h of cold treatment (Fig. 3A,B). In Cavendish Banana, the significant changes were found in abundance of 180 phosphopeptides (Fig. 3C,D, see Supplementary Table S1), of which 48 increased in abundance and 132 were less abundant. In Dajiao, a total of 83 differential phosphopeptides were found (Fig. 3C,D, see Supplementary Table S1), of which 58 were increased in abundance and 25 were less abundant in 3 h cold stress over no stress control.

To compare the number of differentially expressed phosphopeptides after cold stress, all the phosphopeptides found in both Cavendish Banana and Dajiao data sets with differential abundance were shown in Venn diagram (Fig. 3, see Supplementary Table S1). There are 25 phosphopeptides with differential abundance being found in both sets (Fig. 3B). Strikingly, only one phosphopeptide with increased abundance was commonly found in both Dajiao and Cavendish Banana (Fig. 3C) and only 3 phosphopeptides with decreased abundance in both species (Fig. 3D), indicating 21 out of 25 differential phosphopeptides went to opposite direction after cold stress between Dajiao and Cavendish Banana. These results strongly suggest that phosphorylation dynamics of Cavendish Banana in response to cold stress appear fundamentally different from those of Dajiao.

### Interaction Networks of Phosphoproteins Containing Differentially Abundant Phosphopeptides under Cold Stress

**Figure 4:**
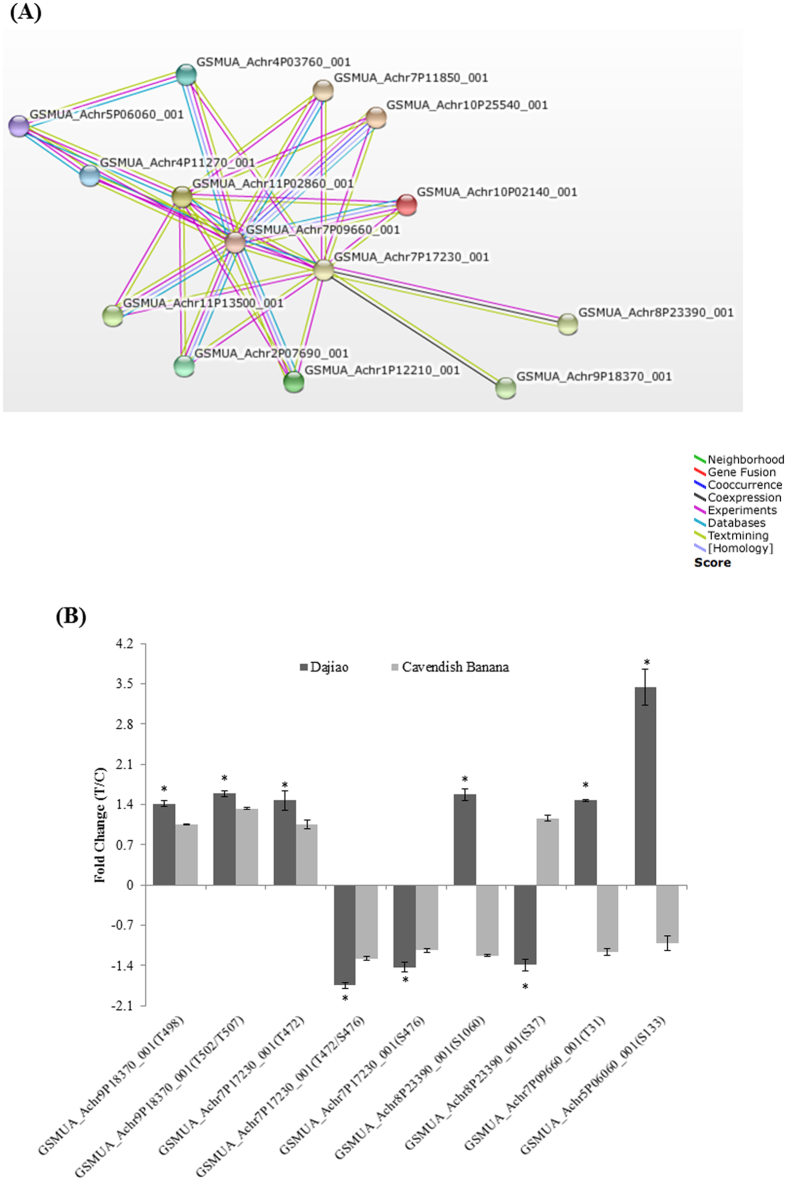
(**A**) Functional interaction network between MAPK cascade and related significantly changed phosphoproteins. Five significantly changed phosphoproteins identified in this study and 8 MAPK cascade proteins were initially submitted to conduct blast searching against existing databases in STRING 10 software. (**B**) Comparison of ratio changes of 9 phosphopeptides with significantly differential abundance between Dajiao and Cavendish Banana, whose proteins were predicted to be involved in MKK2 functional interaction network. *means significantly changed.

Mitogen-activated protein kinase kinase 2 (MKK2), a key member of MAPK cascades, is one of the prominent phosphoprotein found in this work. Since it was reported that phosphorylation of MAPK alters its downstream cascades in *Arabidopsis* cold resistance^7^,^18^, it inspired us to investigate the possible effect of the MKK2 network on the cold tolerance in *Musa spp*. By supplementing six MEKK1 proteins, two MAPK4 proteins and one MAPK6 (see Supplementary Table S4), annotated in the existing *Musa_AB*_PEP_database along with differential phosphoproteins identified in this study, a STRING-based protein network analysis constructed an interaction network consisting of GSMUA_Achr7P09660_001 (Mitogen-activated protein kinase kinase 2) and 4 associated phosphoproteins of interest (Table 2). After filtering with 70% confident score, the final interaction network consists of MKK2 and 4 phosphoproteins provides a convincing evidence that MKK2 interacts with the 4 phosphoproteins discovered in this study (Fig. 4A).

Notably, the changes of these 5 phosphoproteins under cold stress were completely different between Dajiao and Cavendish Banana (Fig. 4B, Table 2). In Dajiao, among the 9 phosphopeptides belonging to the 5 phosphoproteins, 3 phosphopeptides significantly decreased in abundance, and the other 6 phosphopeptides significantly increased (p-value < 0.05). In contrast, none of the counterparts in Cavendish Banana significantly changed in abundance. An expanded view of the interaction network consisting of these 5 identified phosphoproteins and MAPK cascade reveals that MKK2 is located in the center of this network (Fig. 4A). This finding suggests that MKK2 interaction network may be one of the key candidate phosphorylation networks regulating cellular pathways and metabolism responsible for the different cold tolerance between Dajiao and Cavendish Banana.

### Verification of MKK2 Phosphorylation by Western Blotting and Sequence Alignment Analysis

**Figure 5:**
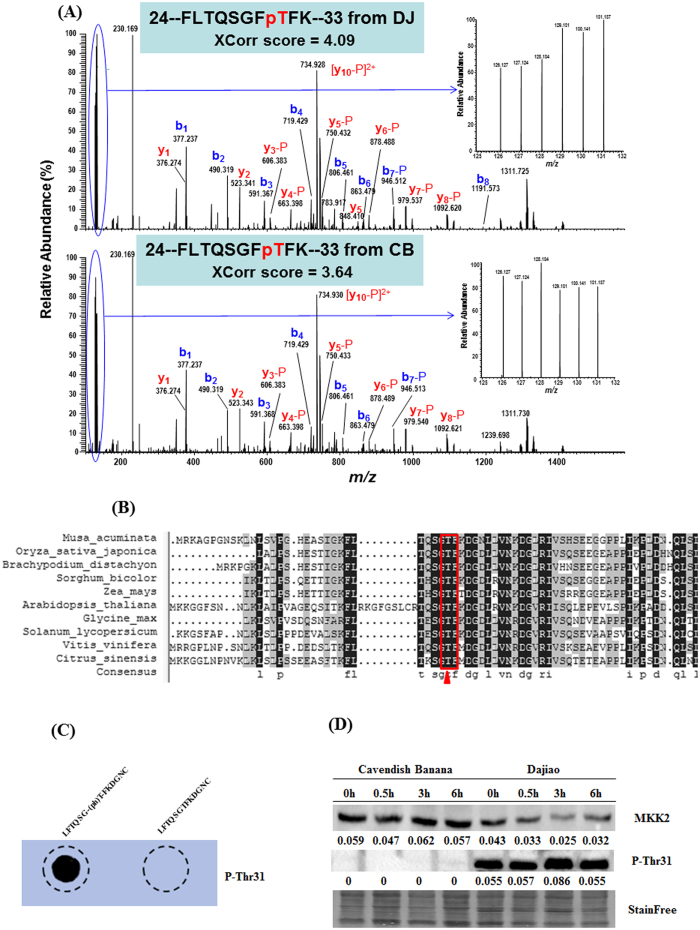
Identification and validation of a T31 phopshopeptide of MKK2 protein in response to cold stress. (**A**) MS/MS Spectra of a doubly-charged ion at m/z 783.919^2+^ confidently identifying a TMT-labeled tryptic peptide with T31 phosphorylation from MKK2 protein (GSMUA_Achr7P09660_001) in both Dajiao (top panel with Xcorr score at 4.09) and Cavendish Banana (bottom panel with Xcorr score at 3.64). The inset of each panel shows the expanded view of relative intensity of 6 TMT reporter ions for determining the abundance changes of the T31 phosphopeptide in response to cold stress at 0 h in triplicate (126, 127, 128) versus 3 h (129, 130, 131). (**B**) Amino acid sequence alignment of MKK2 between *Musa spp*. and nine species indicates that T31 residue is conserved among all important plant species, suggesting the phosphorylation of T31 residue in MKK2 protein may play an important regulatory role in *Musa spp* under cold stress. (**C**) Dot blot of unmodified or phosphorylated MKK2 peptides with anti-MKK2 Thr31 antibody. (**D**) A rabbit polyclonal antibody against MKK2 intact protein and a rabbit polyclonal phospho-specific antibody against p-Thr31 of MKK2. Numbers indicate the western blotting signal intensities normalized to the total protein contents, using a novel Stain-free technology for total protein quantification^72^.

A phosphopeptide containing threonine-31 residue (T31) from MKK2 protein was confidently identified and quantified based on the associated MS/MS spectra as shown in Fig. 5A for both Dajiao (top panel) and Cavendish banana (bottom panel). The relative intensity of 6 TMT reporter ions (Fig. 5A) in the MS/MS spectra showed a 1.47-fold increase in abundance of the T31 phosphopeptide in response to cold stress in Dajiao and a slight decrease of abundance (0.85-fold) found in Cavendish Banana (Table 2).

Sequence alignment of MKK2 protein between *Musa spp*. and nine most common plant species (*Oryza sativa japonica* (Japanese rice); *Brachypodium distachyon*; *Sorghum bicolor* (sorghum); *Zea mays* (maize); *Arabidopsis thaliana* (thale cress); *Glycine max* (soybean); *Solanum lycopersicum* (tomato); *Vitis vinifera* (wine grape); *Citrus sinensis* (orange)) demonstrated that the T31 residue is fully conserved in all ten species (Fig. 5B), suggesting T31 phosphorylation in MKK2 could be important in many other species. Furthermore, the counterpart residues of T31 in both rice^38^ and soybean^27^ were reported to be phosphorylated, providing an additional conformation of T31 as a conserved phosphorylation site in *Musa spp*.

Western blot was also used to validate the abundance changes of MKK2 intact protein and its phosphosite (T31) found from discovery-based phosphoproteomic analysis in Dajiao and Cavendish Banana under cold stress. The embryogenic cell suspension of Dajiao and Cavendish Banana under 0 h, 0.5 h, 3 h and 6 h cold stress were used to extract the proteins for western blot analysis.

Dot blot was used to verify the specificity of phosphor-specific antibody against p-Thr 31 of MKK2 (Fig. 5C). The results showed the phosphor-specific antibody enabled to specifically recognize the synthesized phosphorylated MKK2 peptide (_LFTQSG-(ph)T-FKDGNC_) but not the unmodified equivalent peptide (_LFTQSGTFKDGNC_) of MKK2, confirming the specificity of this antibody.

As shown in Fig. 5D, MKK2 intact protein of Cavendish Banana remained stable with gradually increased expression in the time course of cold stress. However, T31 phosphorylation of MKK2 in Cavendish Banana was not detected by phosphor-specific antibody (Fig. 5D), despite the fact that a 0.85-fold decrease after 3 h cold stress was found in the phosphoproteomics data (Fig. 5A). The difference between western blot result and phosphoproteomics data is likely due to the different source materials, in which the TMT-based phosphoproteomics data were from Cavendish Banana leaves while protein samples for western blotting shown in Fig. 5D came from Cavendish Banana embryogenic cell suspension. Banana leaves contain high abundant proteins and rich in polysaccharides and phenol, which will cause adverse impact on western blot results. Thus, to minimize the interferences from leaves tissues and obtain accurate information about MKK2 protein changes in cells under cold stress, embryogenic cell suspension were used for western blot.

The observation of T31 phosphorylation was found completely opposite in Dajiao, where MKK2 protein at 3 h cold stress was decreased by 0.58-fold compared to 0 h control. More surprisingly, the T31 phosphorylation at 3 h cold stress was significantly increased by 1.56-fold (Fig. 5D) compared to that at 0 h cold stress, which is in good agreement with the data obtained in MS/MS spectrum (1.47-fold, Fig. 5A). These results indicate the change of MKK2 protein under cold stress was completely independent from the abundance change of the MKK2 T31 phosphosite. Given the fact that the MKK2 pathway was reported to mediate cold and salt stress signaling in *Arabidopsis* (18), we suspect that the remarkable difference of MKK2 T31 phosphorylation abundance and its occupancy in Dajiao and Cavendish Banana in response to cold stress may contribute to the higher cold tolerance of Dajiao.

### Sucrose and Trehalose Content under Cold Stress

Sucrose phosphate synthase (SPS) and trehalose-6-phosphate synthase (TPS), which showed a significant increase in the abundance of phosphopeptides in Dajiao under cold stress, were identified and enriched in the sugar metabolism pathway. The abundance of two phosphosites (S153 and S157) in the core peptide: NFSDIQSWSDDEKER of SPS (GSMUA_ Achr4P16070_001), as shown in Table S1, were showed to be significantly increased in Dajiao under cold stress, while in Cavendish Banana only S153 was detected and its abundance remained without a change. To test if the SPS phosphorylation has any effect on the sucrose content under cold stress, we measured the sucrose content of Cavendish Banana and Dajiao in the extended time course of cold stress. The result showed that after 3 h and 6 h of cold stress, the sucrose content in Cavendish Banana steadily increased, while in Dajiao the sucrose content revealed an early burst at 3 h and slight decay at 6 h. A big difference was found at 24 h of cold stress when the sucrose content of Cavendish Banana decreased to nearly normal level, while the sucrose level in Dajiao was steadily increasing (see Supplementary Fig. S5). In this study, a phosphosite S40 of TPS (GSMUA_ Achr8P27770_001) significantly increased in Dajiao under cold stress but no significant changes in Cavendish Banana was observed (see Supplementary Table S1). The profiles of the trehalose content in Cavendish Banana and Dajiao in the time course of cold stress were also determined. The trehalose content in Cavendish Banana initially decreased at 3 h of cold stress, and then rapidly increased at 6 h and 24 h, while the trehalose content in Dajiao was steadily increasing at all three time points (see Supplementary Fig. S5).

## Discussion

All plants have evolved adaptation mechanisms to survive adverse environmental conditions such as suboptimal growth temperatures. In plants, the response and adaptation to cold stress are initiated by complex and rapid signaling pathways that affect a wide range of cellular processes, including transcriptional regulation, cell division and morphogenesis, protein folding and metabolic changes in nutrient flux^39^. Our group has a long-standing research interest in understanding the molecular mechanisms of cold tolerance in *Musa spp*. Comparison of the phosphoproteome data in this study with our previous transcriptional profiling^6^ and quantitative proteomics results^11^ reveals that there is a little correlation between global phosphoproteomes and gene transcription, and low degree of correlation between Dajiao’s proteome and phosphoproteome^11^. There are only a third of the total identified and quantified phosphoproteins that were commonly found in our previous Dajiao proteome data (see Supplementary Fig. S4). In other words, ~67% of total Dajiao phosphoproteins identified in this study in response to cold stress were new identifications. More interestingly, all the important phosphoproteins (such as MKK2, HY5 and STN7 etc.) found in this work have not been identified or quantified in the previous Dajiao proteome data^11^, indicating the majority of low abundant phosphoproteins were indeed missed by global proteome analysis. Our phosphoproteome was measured at 3 h after cold stress while proteome data was collected for 6 h and 24 h after cold stress. The early time point to the cold stress in the global phosphoproteome experiment may contribute to some extent to the successful detection of those unique phosphoproteins, which also supports our hypothesis that phosphorylation regulation happens at early stage, while the other cellular responses appear in relatively late stages. These observations reported here also support the significance of protein phosphorylation as a primary response to cold stress found in several model plants^22^,^40^,^41^. In the present study, the results from a global profiling of comparative phosphoproteomes between Dajiao and Cavendish Banana under cold stress leads us to discover a conserved MKK2 network in *Musa spp*., which appears to be associated with regulation of cellular functions responsible for the high cold tolerance in Dajiao.

MAPK cascades are highly conserved signal transduction modules in animals, plants and yeast^42^. Several lines of evidence suggest that the MAPK signaling cascade is, at several levels, involved in plant biotic or abiotic stress induced signaling and indeed, most plant MAPKs investigated to date have been linked to stress responses^43^,^44^,^45^,^46^. As the nodal point of the MAPK cascades, the roles of MAPKs in response to biotic and abiotic stresses have been intensively investigated. For instance, *Arabidopsis* MKK2 has been shown to be activated by MEKK1 to increase freezing and salt tolerance by activating its direct targets MPK4 and MPK6, as well as the expression of other stress-induced marker genes^18^. ZmMKK4, a novel group C mitogen-activated protein kinase kinase in maize, confers salt and cold tolerance in transgenic *Arabidopsis*^47^ and ZmMKK1 positively regulated the salt and drought tolerance in transgenic *Arabidopsis*^48^.

In this study, the MKK2 phosphorylation on T31 residue, along with 4 other phosphorylated proteins associated with MKK2 interaction network showed a remarkable difference between Dajiao and Cavendish Banana in response to cold stress. From the phosphoproteomic data and western blot results, the T31 phosphorylation level and its occupancy of MKK2 in Dajiao are consistently and significantly increased, suggesting T31 phosphorylation and MKK2 abundance appear specifically responsive to cold stress. In Cavendish Banana, phosphoproteomic data of MKK2 shows slight (0.85-fold) decrease of T31 but no detection in western blotting, which may be due to the combining effects of low abundance T31 in Cavendish Banana embryogenic cell suspension and its extremely low occupancy rate without enrichment. Interestingly, the T31 phosphorylation in MKK2 has been reported in both *Oryza sativa*^38^ and *Glycine max*^27^. However, no functional characterization or cold tolerance data of T31 phosphorylation have been reported. We also found that T31 residue of MKK2 was extraordinarily conserved among *Musa spp*. and nine most common crops and plant species, indicating that T31 phosphorylation could be important in MKK2 protein functions on particularly signaling regulation. The results of T31 phosphorylation and MKK2 profiling between Dajiao and Cavendish Banana lead us to hypothesize that the T31 phosphorylation could be associated with regulation of signaling pathways or phosphorylation network for the subsequent cellular functions for high cold tolerance in Dajiao. An *in vivo* salvage experiment using *Arabidopsis* transgenic lines for overexpression of MMK2 and its T31 mutant for testing the hypothesis is underway.

In the MKK2 interaction network found in this work, every phosphopeptide showed a considerably different expression pattern between Dajiao and Cavendish Banana in response to cold stress. Transcription factor HY5 is a bZIP transcription factor that has a pivotal role in light signaling, mediating photoreceptor responses to promote photo morphogenesis, and it has also been described to mediate plant responses to UV-B and different hormones, such as ABA, gibberellins, cytokinin, and auxins^49^,^50^. In *Arabidopsis* HY5 levels are regulated by low temperature transcriptionally via a CBF- and ABA-independent pathway, and post translationally via protein stabilization through nuclear depletion of COP1. HY5 also positively regulates cold-induced gene expression through the Z-box and other cis-acting elements, ensuring the complete development of cold acclimation^51^. Our phosphoproteomic data contain a phosphopeptide of HY5 (GSMUA_ Achr5P06060_001) at Ser133, which has also been reported in *Arabidopsis*^30^. In the MKK2 interaction network of *Musa spp*. (Fig. 4), HY5 appears to interact with MAPK6 (GSMUA_Achr11P02860_001), which was reported to be phosphorylated by MKK2 mediating cold and salt stress signaling in *Arabidopsis*^18^. The abundance of S133 phosphorylated peptide in HY5 phosphoprotein of Dajiao increased by 3.44-fold at 3 h cold stress while no significant change found in Cavendish Banana, strongly suggesting S133 phosphorylation of HY5 protein within MKK2 network found in Dajiao could play a role in its cold resistance.

The second phosphoprotein in MKK2 network is calcium-sensing receptor (CaSR). It is now known that alterations in extracellular calcium concentration in a variety of tissues exert diverse physiological effects mediated by a CaSR^52^, which is related to both stomatal movement and photosynthetic electron transport, crucial for water use efficiency and drought tolerance in *Arabidopsis*^53^. Recently Tomoyuki Furuya’s group reported Ca^2+^ signaling occurred upstream of the MEKK1–MKK2 pathway^24^, and showed that CaSRs played an important role in plant resistance to abiotic stress. In our phosphoproteomic data (Table 2), we detected two phosphopeptides from CaSR (GSMUA_Achr9P18370_001) whose abundance were significantly increased after cold stress in Dajiao but no significant changes in Cavendish Banana. Sequence alignment indicates that both, singly phosphorylated peptide at T498 and a doubly phosphorylated peptide at T502/T507, of CaSR were conserved among the most common plants, but no phosphorylation on those sites has been found in other species. In MKK2 network, CaSR interacts with STN7 (GSMUA_Achr7P17230_001), which has been found to interact with MKK2. These findings suggest that cold stress induces changes in the cellular calcium content, and the change of Ca^2+^ will interact with MAPK cascades that may further affect the downstream proteins through phosphorylation and lead to high cold stress tolerance in Dajiao.

Another kinase found in this network, Serine/threonine-protein kinase STN7, chloroplastic (STN7) (GSMUA_Achr7P17230_001), was phosphorylated on two sites (T472 and S476). STN7 has been proposed to be a conventional signaling kinase, instigating the phosphorylation cascade required for coordinated expression of photosynthesis genes, assembly of the photosynthetic machinery, control of redox balance in the electron transfer chain, and STN7 is affected by temperature^54^,^55^. T472 phosphorylation, which has been detected in *Arabidopsis*^30^ and *Medicago truncatula*^56^, significantly increased by 1.47 fold in Dajiao, and S476, which has also been found in *Arabidopsis*^30^ and *Medicago truncatula*^56^, significantly decreased by 0.7 fold in Dajiao. In contrast, both were not significantly changed in Cavendish Banana under cold stress (Table 2). The opposite changes in abundance of the STN7’s two phosphorylated sites with proximity location in its primary sequence indicate that enzymatic activity and phosphorylation status of STN7 may play complex regulatory roles in the MKK2 network for Dajiao in response to cold stress. Significant decrease in abundance (0.57-fold) of the doubly phosphorylated peptide (T472/S476) found in Dajiao, as shown in Table 2, indicates that S476 phosphorylation is likely to have more profound effect in the network than T472. Further studies are needed to characterize the functions of T472 and S476 phosphorylation.

The S1060 phosphosite of kinesin-like protein KCA2 (GSMUA_Achr8P23390_001) was significantly increased in Dajiao under cold stress, while no significant change found in Cavendish Banana. Phosphorylation on S37 of KCA2 however, was significantly decreased in Dajiao under cold stress, while no change remained in Cavendish Banana. KCA2 was reported to play critical roles in mitosis, morphogenesis, signal transduction, and regulating gibberellin biosynthesis and cell growth by transcription activation activity^57^. S1060 phosphosite equivalent has also been found in *Arabidopsis*^38^, *Oryza sativa*^38^ and *Vitis vinifera*^58^, suggesting that the S1060 phosphosite is of relatively higher evolutionary conservation in different plant species. However, S37 is conserved among the most common plants while the phosphorylation has not been reported in other plants.

The findings from the present study combined with the previous relevant studies support that MKK2 interaction network plays a critical role in response to cold stress in several model plants and we believe the MKK2 network we found here plays an important role in cold tolerance of Dajiao.

SPS is considered to be a primary regulator for controlling biosynthesis and accumulation of sucrose, and plays an important role in plant viability^59^,^60^. SPS phosphorylation has been reported on multiple Ser residues^25^,^61^. Sucrose has recently been recognized to have important hormone-like functions as a primary messenger in signal transduction. Additionally, sucrose molecules regulate gene expression at both transcriptional and post-transcriptional levels^62^,^63^. A previous study demonstrated that low temperature can selectively inhibit SPS activity and therefore reduce starch to sucrose conversion in banana fruit during ripening^64^. These findings suggest that cold stress may affect the enzymatic activity of phosphorylated SPS or other enzymes relevant to sucrose metabolism. We speculate that the different response of SPS phosphoprotein to cold stress in Dajiao and Cavendish Banana is one of the factors that explain completely different profiles of the sucrose content particularly at the extended time of cold stress.

Trehalose plays an important role in metabolic regulation and abiotic stress tolerance in a variety of organisms. In plants, its biosynthesis is catalyzed by two key enzymes: trehalose-6-phosphate synthase (TPS) and trehalose-6-phosphate phosphatase (TPP)^65^. In our research results trehalose content suggests the increased phosphorylation of TPS at 3 h cold stress in Dajiao that may affect the enzymatic activity of TPS or relevant enzymes involved in the trehalose metabolic pathway, which, in turn, enables the accumulation of trehalose at the early stage of cold stress that was found in Dajiao. Taken together, the data support the theory that the phosphorylation of key enzymes involved in sugar metabolism pathway may play an important regulatory role in sugar metabolism, which is ultimately attributed to different cold tolerance between Dajiao and Cavendish Banana.

**Figure 6:**
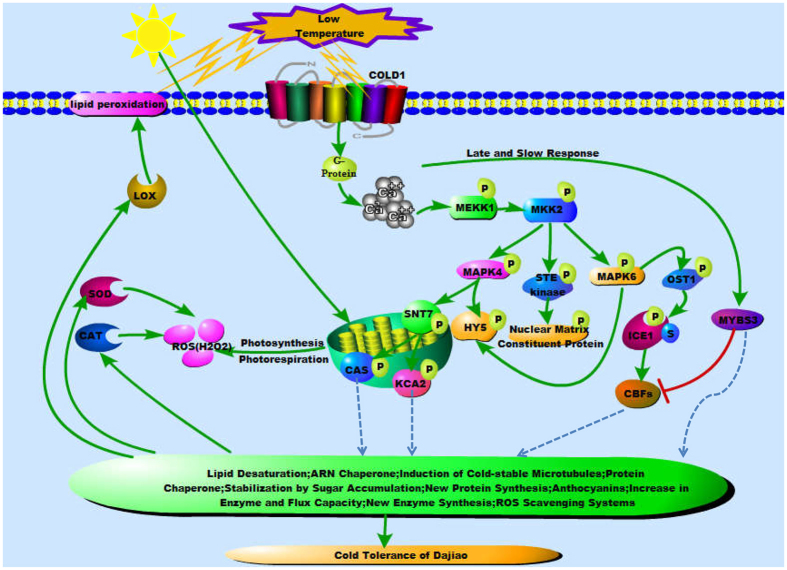
A diagrammatic mode illustrating the putative cold-tolerance network in cold-resistant Dajiao based on our previous publications on global proteomics: Yang *et al*.^11^, global transcriptomics: Yang *et al*.^6^ as well as the information gained in this global phosphoproteomic study. Broken arrows indicate indirect regulation; solid arrows indicate activation, whereas lines ending with a bar show negative regulation; P, phosphorylation; S, SUMO.

Our study, using TMT-based quantitative phosphoproteomic analysis, represents the first comprehensive phosphoproteomic analysis of cold-sensitive Cavendish Banana and cold-tolerant Dajiao in response to cold stress. In combination of the results acquired from this work and previous studies, we propose an expanded model towards a cold tolerance mechanism by MMK2 signaling pathway as illustrated in Fig. 6. In early stage of cold stress in Dajiao, COLD1 interacts with G protein to activate the Ca^2+^ channel for temperature sensing^66^, which initially stimulates MAPK cascades. The phosphorylation of MAPK cascades triggers the activation of related protein kinases (*e.g*. OST1^22^, STN7) and other phosphoproteins (*e.g*. CAS, ICE1^6^). Subsequently, a series of physiological and biochemical reactions directly (e.g. ROS Scavenging systems^11^) or indirectly associated with Dajiao cold tolerance begin to play their regulatory roles. In later stage of cold stress, expression of MYBS3 begins to recover^6^ and regulates multiple genes of cold response (Fig. 6). While in cold-sensitive Cavendish Banana, no such corresponding regulations were observed. The next questions that need to be answered are how to demonstrate that T31 of MKK2 is involved in regulation of signaling pathways and phosphorylation network for the subsequent cellular functions, and is responsible for high cold tolerance in Dajiao? How do the phosphoproteins in MKK2 interaction network coordinate together to effectively regulate cold resistance? Addressing these questions will not only further enhance the understanding of regulation of phosphorylation of cold tolerance in Dajiao, but also will be conducive to nurturing new varieties of cold tolerant banana cultivars.

## Materials and Methods

### Plant Material, Stress Treatment and Experimental Design

Seedlings of the cold-tolerant Dajiao (*Musa spp*. Dajiao; ABB Group) and the cold-sensitive Cavendish Banana (*Musa spp*. Cavendish; AAA Group) with a uniform growth stage were obtained from Institute of Fruit Tree Research, Guangdong Academy of Agricultural Sciences, Guangzhou, P. R. of China. Seedlings were grown in a growth chamber under a 30/28 °C (day/night) temperature regime, a photon flux density of 240 μmol m^−2^ s^−1^ throughout a 12 h photoperiod, and a relative humidity of 60–80%. Five-leaf stage seedlings were used in the experiment. Low temperature treatments were operated according to Yang *et al*.^6^.

### Measurements of Photosynthetic Rate, Conductance to H_2_O, Intercellular CO_2_ Concentration and Transpiration Rate

Leaf net photosynthetic rates, conductance to H_2_O, intercellular CO_2_ concentration and transpiration rates were measured using a portable gas analysis system, LI-COR 6400 with LED light source (LICOR Inc., Lincoln, NE). The measurement conditions were set as followed: leaf temperature 25 °C, photon flux density 800 μmol·m^−2^ s^−1^, humidity 65%, and CO_2_ concentration 0.038%.

### Measurements of Sucrose and Trehalose Content

The first young leaf of Dajiao and Cavendish Banana was detached from the top of each of the 5 plants at four time points (10 °C for 0 h, 3 h, 6 h and 24 h) for each biological replicate. Sucrose content was measured according to a Sucrose Content Kit (spectrophotometry, Cominbio, Suzhou, China). Trehalose content was measured by a Trehalose Content Kit (spectrophotometry, Cominbio, Suzhou, China). The experimental design conducted in this study included a total of three independent experiments as biological replicates.

### Protein Extraction, Digestion, and TMT Labeling

Leaves’ crude proteins were isolated and purifed as described by Isaacson *et al*.^67^, with modifications. The protein concentration for each sample was determined by Bradford assay^68^ using BSA as a standard, and further quantified by running on a precast NOVEX 12% Tris/Glycine mini-gel (Invitrogen, Carlsbad, CA) along with a series of amounts of *E. coli* lysates (2, 5, 10, 20 μg/lane). The SDS gel was visualized with colloidal Coomassie blue stain (Invitrogen), imaged by Typhoon 9400 scanner followed by analysis with ImageQuant Software version TL 8.1 (GE Healthcare).

Further processing of the proteins was then performed according to Thermo Scientific’s TMT Mass Tagging Kits and Reagents protocol (http://www.piercenet.com/instructions/2162073.pdf) with a slight modification.

### Sequential Enrichment of Phosphopeptides by Fe^3+^-IMAC and TiO_2_

The IMAC enrichment was carried out using a PHOS-Select Iron Affinity Gel (Sigma P9740) kit following the vendor-recommended procedure. Specifically, The TMT 6-plex tagged tryptic peptides were reconstituted in 400 μL of Equilibration/Wash Solution (250 mM acetic acid in 30% acetonitrile) and its pH was adjusted to 2.5 with 1 N HCl. The Affinity Gel slurry was added to two spin columns (each with 100 μL of resin) which were rinsed with the Wash Solution. After rinsing the spin columns, 200 μL of samples were added to each spin column with vortex at 1,900 rpm for 60 min. After centrifugation at 10,000 rpm for 30 sec, the unbound fractions were saved, and the spin columns were washed with 200 μL of Wash Solution. The unbound fractions from wash step were saved and pooled for subsequent TiO_2_ enrichment. Following an additional wash with 500 μL of distilled water, the spin columns were eluted with 400 μL of elution buffer (150 mM ammonium hydroxide in 25% acetonitrile) and vortexed at 1900 rpm for 5 min. The eluated material was dried down and stored at −20 °C until subsequent analysis. TiO_2_ enrichment was conducted using a TiO_2_ Mag Sepharose kit (from GE Healthcare). The IMAC-unbound fractions were dried down and reconstituted in 400 μL of binding buffer (1 M glycolic acid in 80% acetonitrile, 5% TFA). The TiO_2_ slurry (75 μL) was used and incubated with the sample for 30 min at 1,800 rpm vortex. After washing the beads with washing buffer (80% acetonitrile, 1%TFA), the phosphopeptides were eluted with 100 μL of elution buffer (5% ammonium hydroxide) twice. The eluted fraction was dried and reconstituted in 25 μL of 0.5% formic acid (FA) for subsequent nano scale LC-MS/MS analysis.

### NanoLC- MS/MS Analysis

The nanoLC-MS/MS analysis was carried out using an Orbitrap Elite (Thermo-Fisher Scientific, San Jose, CA) mass spectrometer equipped with a nano ion source according to Yang *et al*.^11^.

### Data Processing, Protein Identification and Data Analysis

The resulting MS and MS/MS data were processed using MaxQuant (Max Planck Institute of Biochemistry, Munich, Germany) with integrated Andromeda search engine (v.1.4.1.2) and Proteome Discoverer v. 1.4 software (Thermo Fisher Scientific, Bremen, Germany). MS/MS spectra were searched against *Musa_AB*_ PEP_database (43,886 sequences). Trypsin/P was specified as cleavage enzyme allowing up to 2 missing cleavages, 5 modifications per peptide and up to 5 charges. Mass error was set to 20 ppm for first search, 5 ppm for main search and 0.02 Da for fragment ions. Carbamidomethylation on Cys was specified as fixed modification and oxidation on Met, phosphorylation on Ser, Thr, Tyr and acetylation on protein N-terminus were specified as variable modifications. False discovery rate (FDR) thresholds for protein, peptide and modification site were specified at 1%. Minimum peptide length was set at 7. All the other parameters in MaxQuant were set to default values. The site localization probability was set as >0.75 in MaxQuant. To confidently localize phosphorylation sites, the phosphoRS 3.0 node integrated in PD 1.4 workflow was also used to cross-validate the results of MaxQuant. The algorithm of phosphoRS 3.0 software enables automated and confident localizaion of phosphosites by determining individual probability values for each putatively phosphorylated site within the validated peptide sequences^69^.

The distribution charts for assessing raw MS data quality is shown in Fig. S1. The distribution of mass error is around zero and within 5 ppm indicating the mass accuracy of the MS data fits the requirement (Fig. S1). The length of amino acid residues in most peptides distributed between 8 and 20, which correlates with the property of tryptic peptides (Fig. S1), indicating sample digests are satisfactory.

To compare the results for Dajiao’s proteome and phosphoproteome, the previous MS and MS/MS raw data for Dajiao proteome^11^ were re-searched against the same *Musa_AB*_PEP_database using Mascot 2.5 search engine.

### Bioinformatics and Motif Analyses

Gene Ontology (GO) annotation phosphoproteome was derived from the UniProt-GOA database (www. http://www.ebi.ac.uk/GOA/). Meanwhile, sequences of identified proteins were aligned with KOG protein database. After, results of alignment were filtered with parameters: E value < 1e-5, identities >80%, percent of match length >60%, identified proteins’ domain functional description was annotated by InterProScan (a sequence analysis application) based on protein sequence alignment method, in which the InterPro (http://www.ebi.ac.uk/interpro/) domain database was used. For pathway enrichment analysis, the differentially expressed phosphorylated proteins were mapped to the terms in the KEGG (Kyoto Encyclopedia of Genes and Genomes, http://www.kegg.jp/) database using the KOBAS 2.0 (KEGG Orthology-Based Annotation System) program. KEGG pathways with corrected p values ≤ 0.05 were considered to be statistically enriched in cold stress. A subcellular localization predication tool, WoLF PSORT was used to predict subcellular localization. An updated version of WoLF PSORT, PSORT/PSORT II was used for prediction of eukaryotic sequences.

Motif-x software tool was used to create relative frequency plots of the phosphorylation sequences in all identified phosphopeptides. All the database protein sequences were used as a background database, as well as default settings for other parameters as determined by the program.

Phosphoproteins, which phosphopeptide abundances were determined to be differentially expressed in this study, were initially conducted for BLAST search against existing databases in the STRING software, followed by STRING analysis for those BLAST-matched sequence entries to detect potential interactors based on existing database, high throughput experimental data, and text mining^70^. Only interaction networks with the medium confidence (score > 0.7) were reported in this study.

### Western Blot-based Validation Analysis

Total proteins of Cavendish Banana and Dajiao embryogenic suspension cell (ESC) lysate were prepared in ice-cold sodium phosphate buffer (100 mM, pH 7.5) containing 1 mM EDTA, 100 ug/mL PMSF, and 0.1% Triton X-100 with protease/phosphatase inhibitor cocktails (Roche). Equal amount of proteins was subjected to electrophoresis on 15% Criterion polyacrylamide gels (Bio-Rad) under reducing conditions and transferred to polyvinylidene difluoride (PVDF) membranes (Bio-Rad). Total protein amount was detected by imaging in stain-free gels (Bio-Rad). Membranes were then blocked for 1 h with 5% nonfat dry milk (NFM) in Tris-buffered saline (TBS) (pH 7.5) containing 0.05% (wt/vol) Tween 20 (TBST). The membranes were washed three times in TBST and probed over night with the following primary antibodies: polyclonal anti-MKK2 (Abmart Inc. Shanghai, China) at 1:1,000 dilution and polyclonal anti-MKK2 (P-T31) (PTM BioLabs Inc. Hangzhou, China) at 1:20,000. Following primary antibody incubation, the membranes were incubated for 1 h with horseradish peroxidase conjugated sheep anti-rabbit IgG (1:20,000; Sigma, St. Louis, MO). Both primary and secondary antibodies were used diluted in TBST containing 5% NFM. Immunoreaction was detected using an enhanced chemiluminescence (ECL) detection kit (Bio-Rad). The images were obtained by ChemiDoc MP (Bio-Rad) using Image Lab 4.1 software (Bio-Rad). The quantification of blots was performed using Image Lab 4.1.

### Data Availability

The mass spectrometry proteomics data have been deposited to the ProteomeXchange Consortium via the PRIDE^71^ partner repository with the dataset identifier PXD004177.

## Additional Information

**How to cite this article**: Gao, J. *et al*. Comparative Phosphoproteomics Reveals an Important Role of MKK2 in Banana (*Musa spp.*) Cold Signal Network. *Sci. Rep.* **7**, 40852; doi: 10.1038/srep40852 (2017).

**Publisher's note:** Springer Nature remains neutral with regard to jurisdictional claims in published maps and institutional affiliations.

## Supplementary Material

Supplementary Figures and Tables

Supplementary Dataset 1

Supplementary Dataset 2

## Figures and Tables

**Table 1:** Number of phosphoproteins, phosphopeptides, and phosphorylated sites identified in Cavendish Banana and Dajiao under cold stress.

Category	Cavendish Banana	Dajiao
Identified/quantified phosphoproteins	529/352	207/144
Identified/quantified phosphopeptides	679/438	241/167
Identified/quantified phosphorylated sites	772/483	271/188
Phosphopeptides (single/double/multiple)	586/88/5	213/27/1
Phosphorylated sites (on Ser/Thr/Tyr)	653/116/3	209/57/5

**Table 2:** Summary of changed phosphosites in abundance from the proteins associated with MKK2 interaction network.

Protein accession	Position	Modified sequence	PhosphoRS site probabilities %	3 h/0 h Ratio in Dajiao	3 h/0 h Ratio in Cavendish Banana	Reported in other plants
GSMUA_Achr9P18370_001	T498	_FGpTTSSTALQSTRK_	98	1.41 ± 0.05	1.05 ± 0.012	none
GSMUA_Achr9P18370_001	T502/T507	_FGTTSSpTALQSpTRK_	94.5/97.2	1.59 ± 0.05	1.33 ± 0.02	none
GSMUA_Achr7P17230_001	T472	_IIKpTLNESMDELNR_	100	1.47 ± 0.17	1.05 ± 0.07	*Medicago truncatula* (56); *Arabidopsis thaliana* (30)
GSMUA_Achr7P17230_001	T472/S476	_IIKpTLNEpSMDELNR_	100/100	0.57 ± 0.05	0.78 ± 0.03	*Medicago truncatula* (56); *Arabidopsis thaliana* (30)
GSMUA_Achr7P17230_001	S476	_TLNEpSMDELNR_	100	0.70 ± 0.08	0.88 ± 0.04	*Medicago truncatula* (56); *Arabidopsis thaliana* (30)
GSMUA_Achr8P23390_001	S1060	_EEGGpSPIRNPSTAAEDAR_	100	1.57 ± 0.10	0.82 ± 0.02	*Vitis vinifera* (58); *Oryza sativa* (38); *Arabidopsis thaliana* (38);
GSMUA_Achr8P23390_001	S37	_RLpSVSQSSLAPR_	100	0.72 ± 0.10	1.16 ± 0.05	none
GSMUA_Achr7P09660_001	T31	_FLTQSGpTFK_	100	1.47 ± 0.02	0.85 ± 0.06	*Oryza sativa* (38); *Glycine max* (27)
GSMUA_Achr5P06060_001	S133	_RGSSGSATADpSQ_	100	3.44 ± 0.32	0.977 ± 0.13	*Arabidopsis thaliana* (30)
