# Supplementary material for: Comparative Phosphoproteomics Reveals an Important Role of MKK2 in Banana (Musa spp.) Cold Signal Network
Source: Sci Rep. 2017 Jan 20;7:40852. doi: 10.1038/srep40852 (PMC5247763; doi:10.1038/srep40852)
Supplement: Supplementary Figures and Tables [file srep40852-s1.pdf]

Comparative Phosphoproteomics Reveals an Important Role of MKK2 in Banana (*Musa spp.*) Cold Signal Network

Jie Gao [1,2,3,4,*], Sheng Zhang[6,*], Wei-Di He[2,3,4,5], Xiu-Hong Shao[2,3,4], Chun-Yu Li[2,3,4], Yue-Rong Wei[2,3,4], Gui-Ming Deng[2,3,4], Rui-Bin Kuang[2,3,4], Chun-Hua Hu[2,3,4], Gan-Jun Yi[2,3,4] and Qiao-Song Yang[2,3,4]

Supplemental Figure S1a

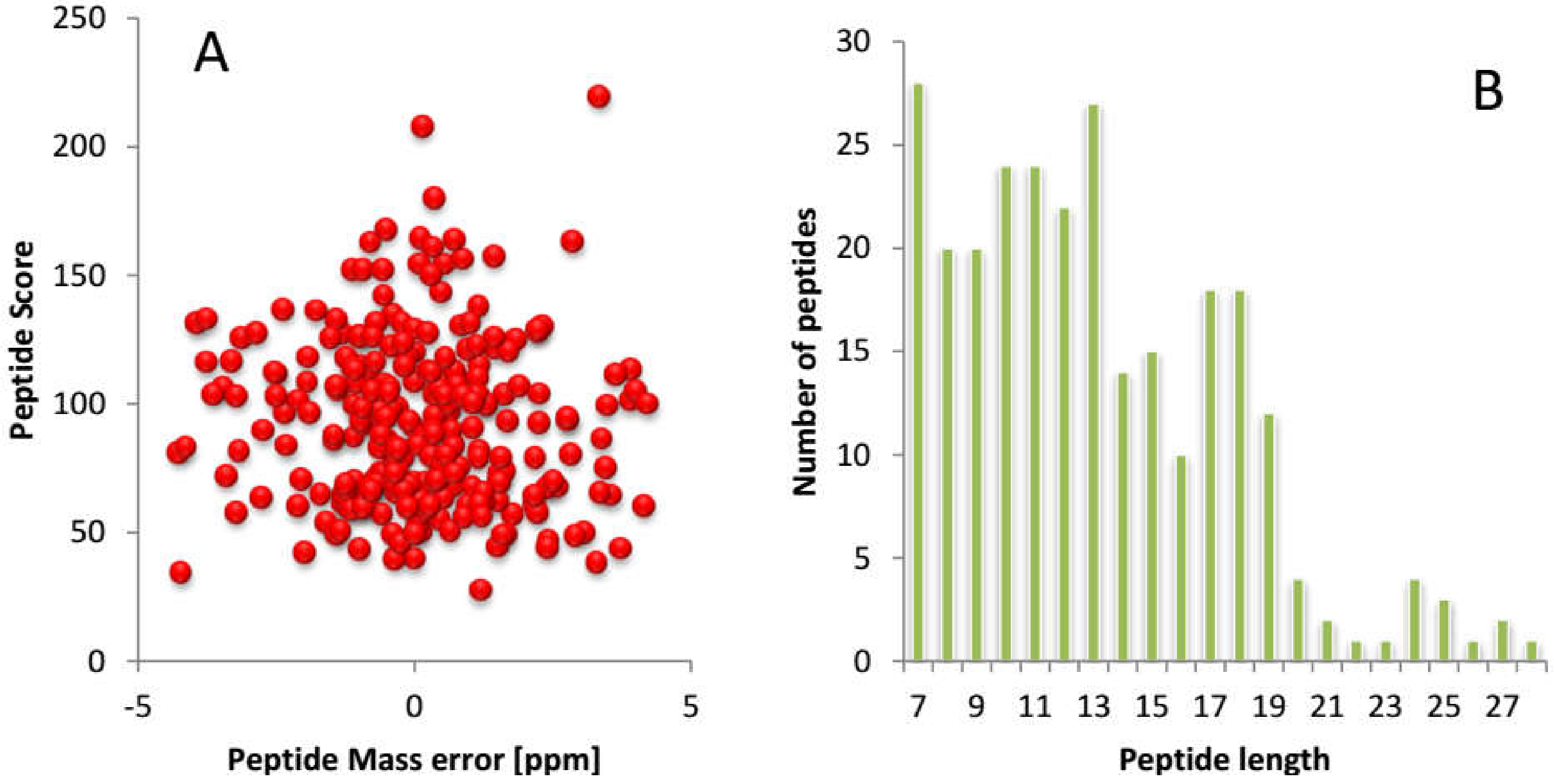

Supplemental Figure S1a. QC validation of Dajiao MS data. (A) Mass error and peptide score distribution of all identified peptides. (B) Peptide length distribution.

Supplemental Figure S1b

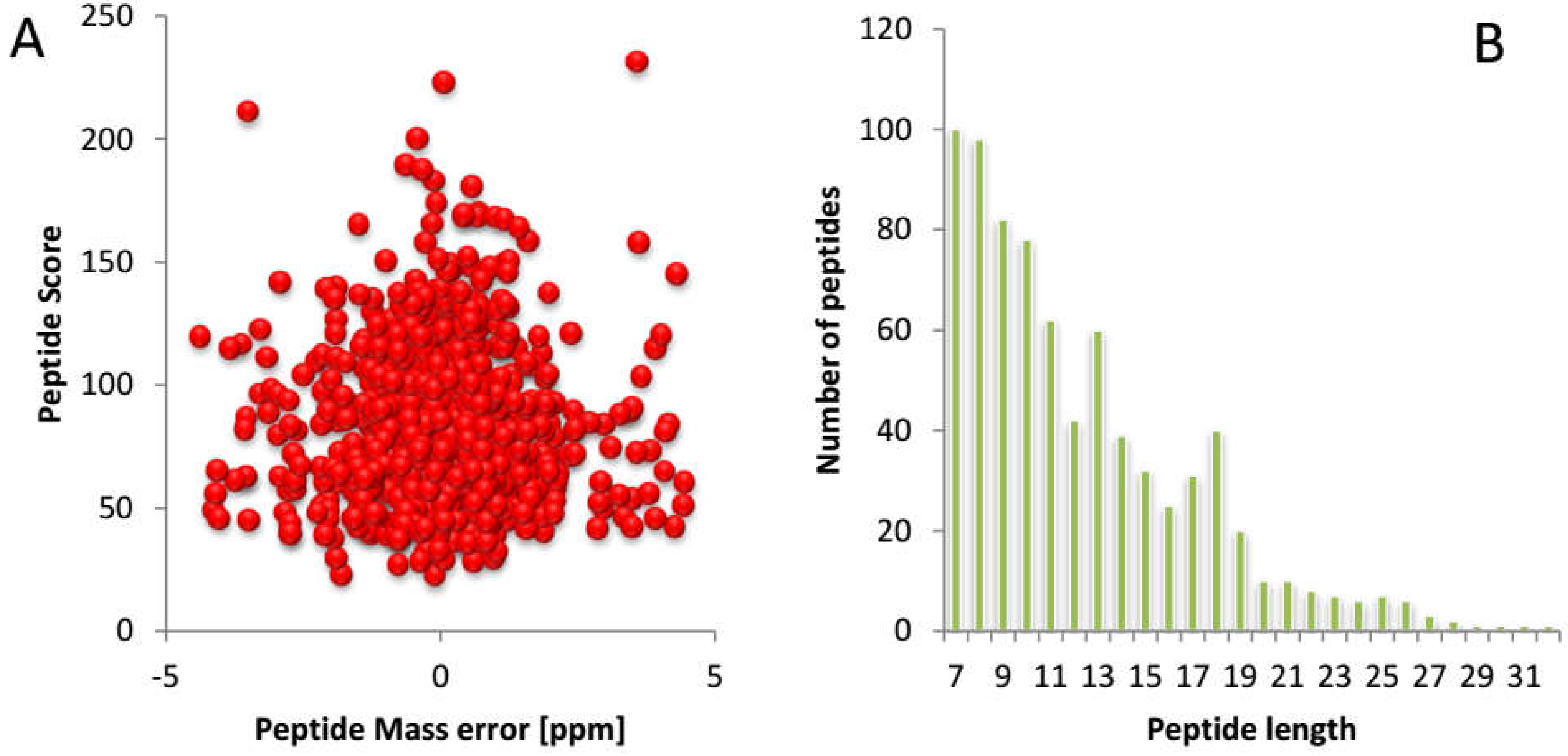

Supplemental Figure S1b. QC validation of Banana Cavendish MS data. (A) Mass error and peptide score distribution of all identified peptides. (B) Peptide length distribution.

Supplemental Figure S2A

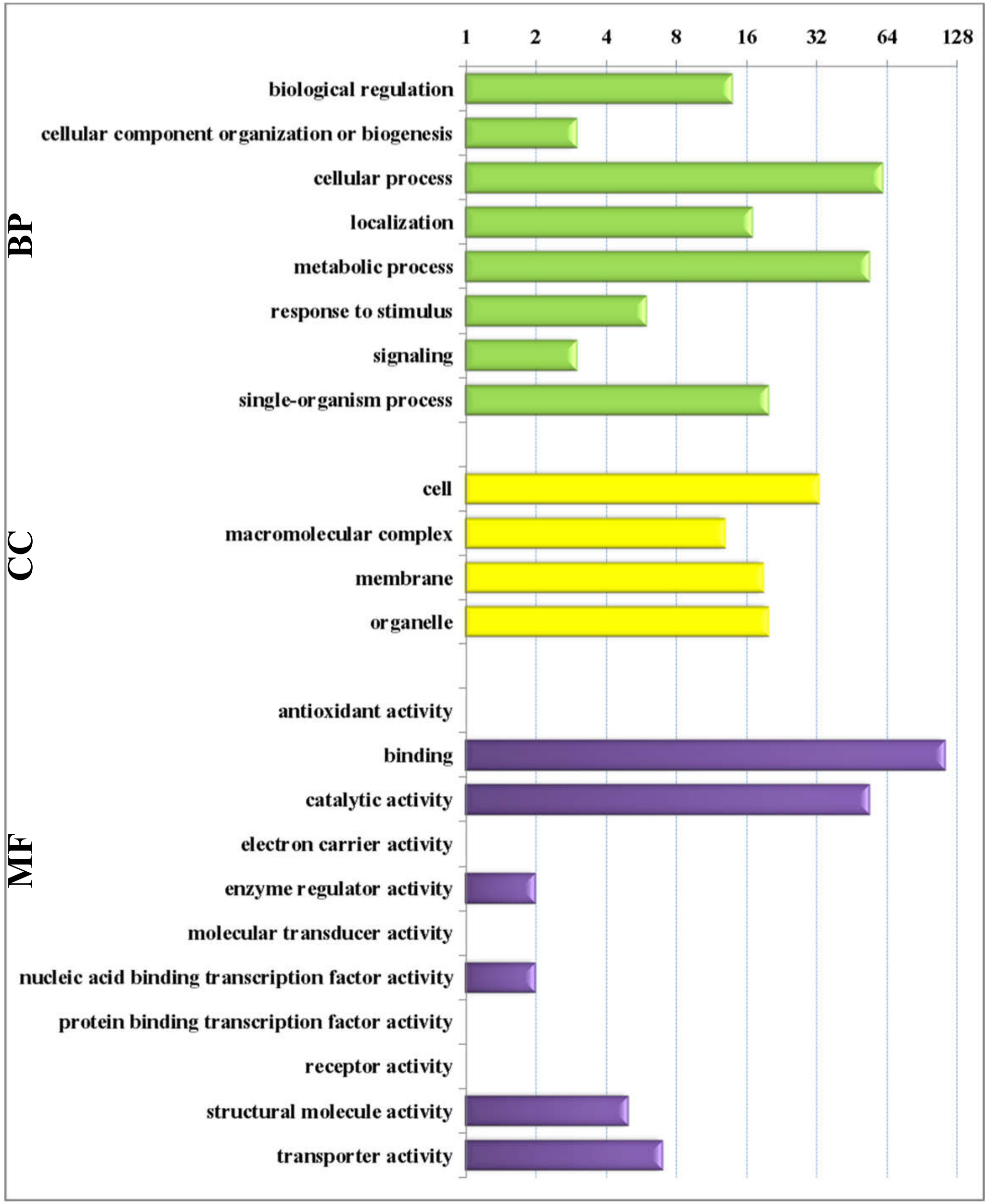

Supplementary Figure S2A.Distribution of phosphoprotein Gene Ontology functional classifications in Dajiao.

Supplemental Figure S2B

Supplementary Figure Distribution of phosphoprotein Gene Ontology functional classifications in Cavendish Banana. BP: biological process, CC: Cellular component, MF: molecular function.

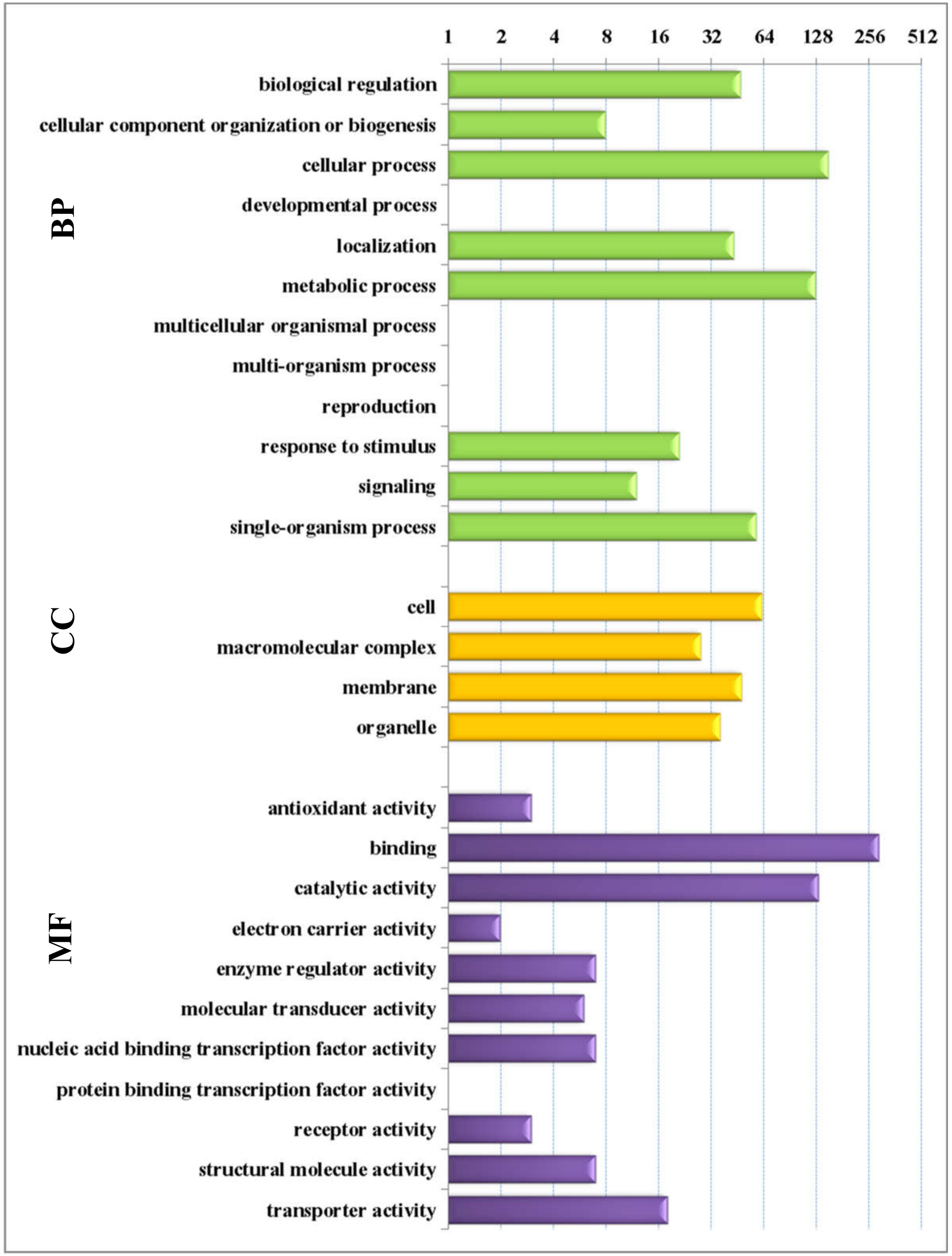

Supplemental Figure S3A

Supplementary Figure S3A. Comparison of $\log_2$ TMT ratio for phosphosites identified in three biological replicates of Dajiao. The largest internal errors were 0.44, corresponding to a 1.35-fold change, which was used as a threshold of significant changes in response to cold stress.

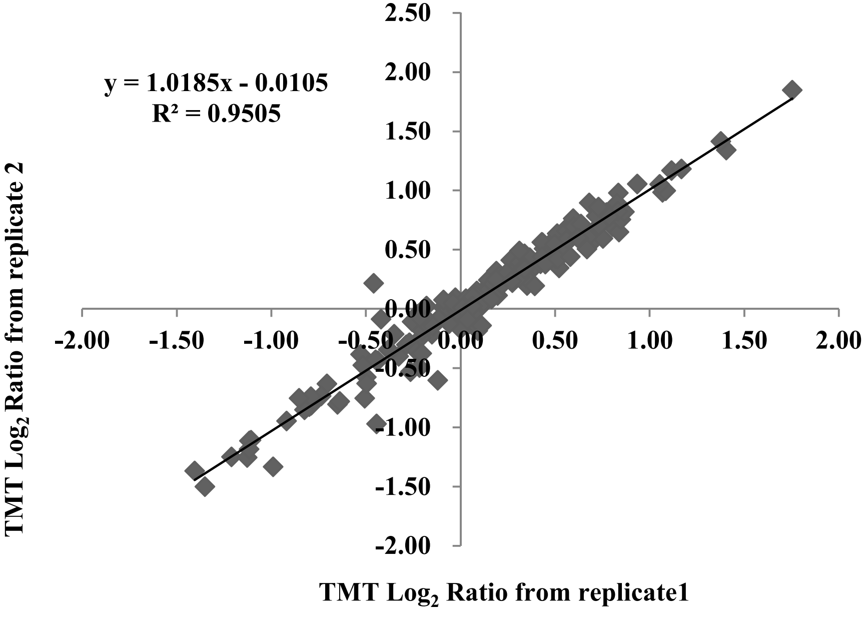

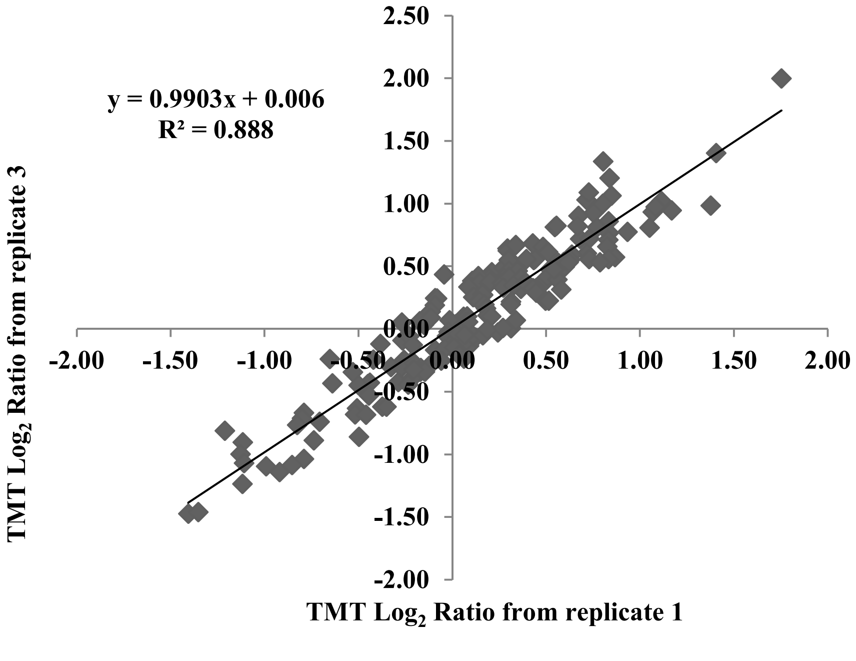

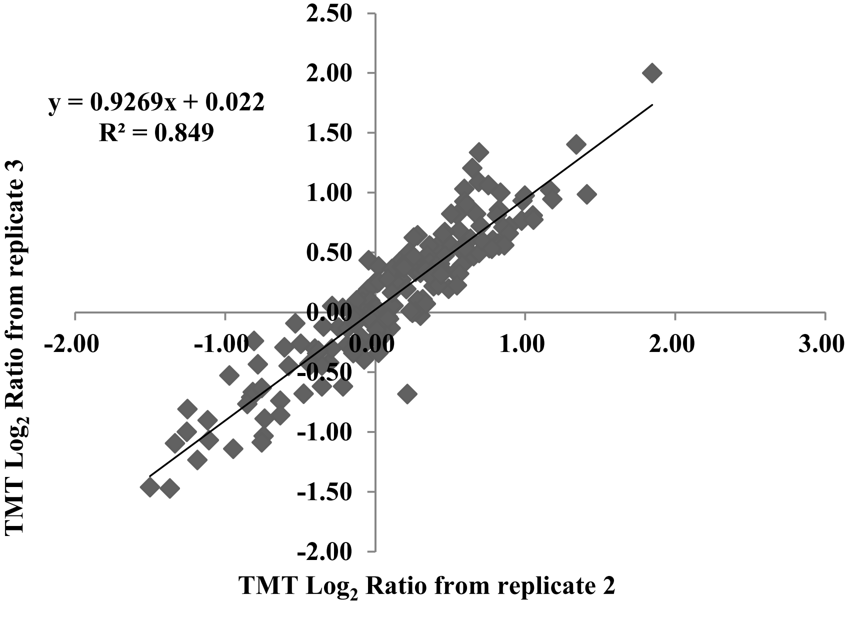

Supplemental Figure S3B

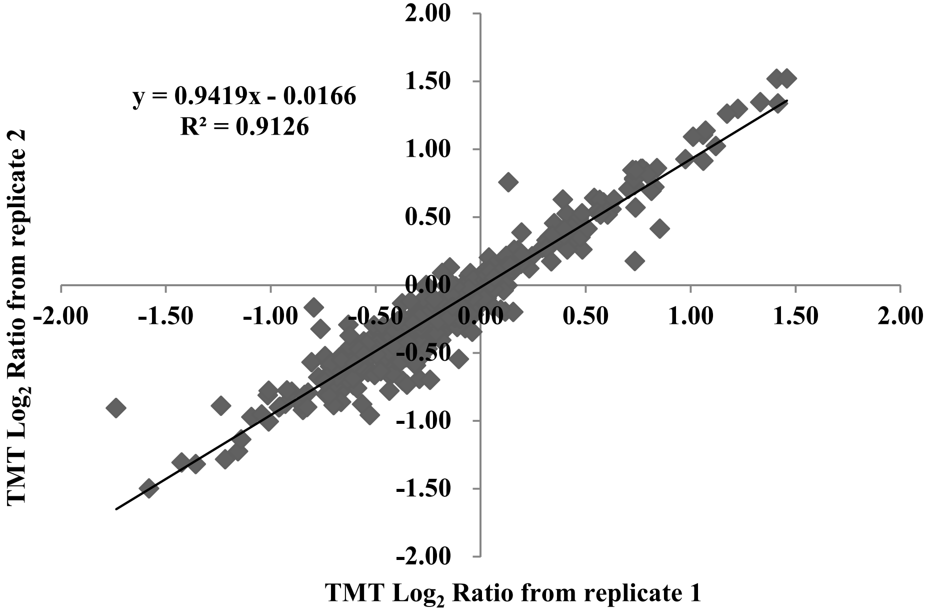

Supplementary Figure S3B. Comparison of log2 TMT ratio for phosphosites identified in three biological replicates of Cavendish Banana. The largest internal errors were 0.42, corresponding to a 1.34-fold change, which was used as a threshold of significant changes in response to cold stress.

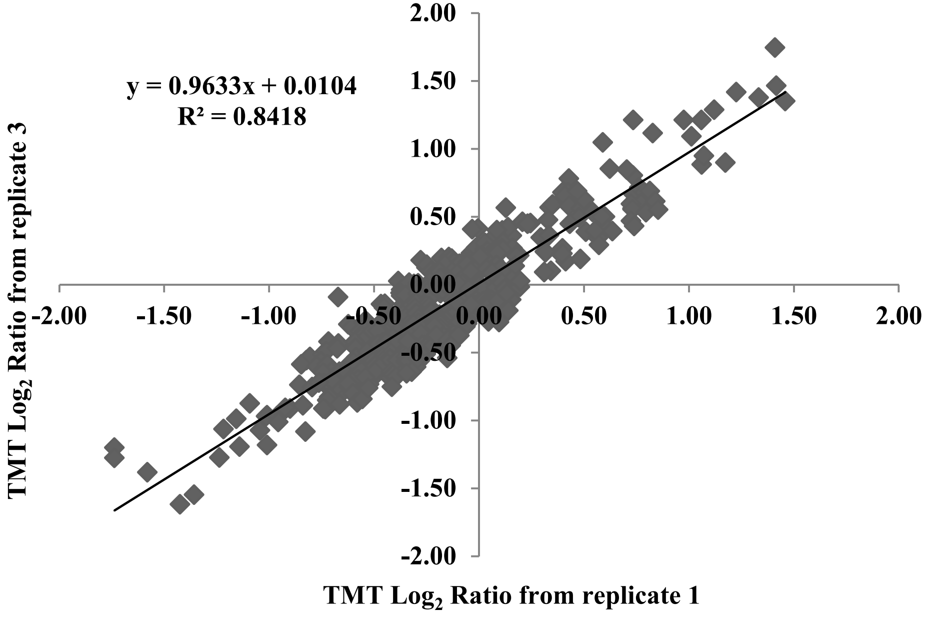

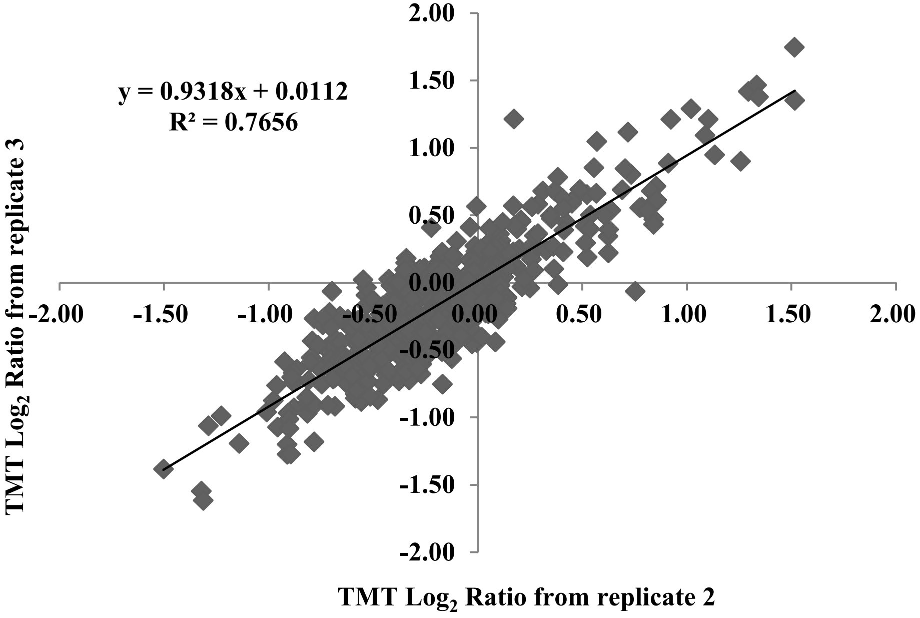

Supplemental Figure S4

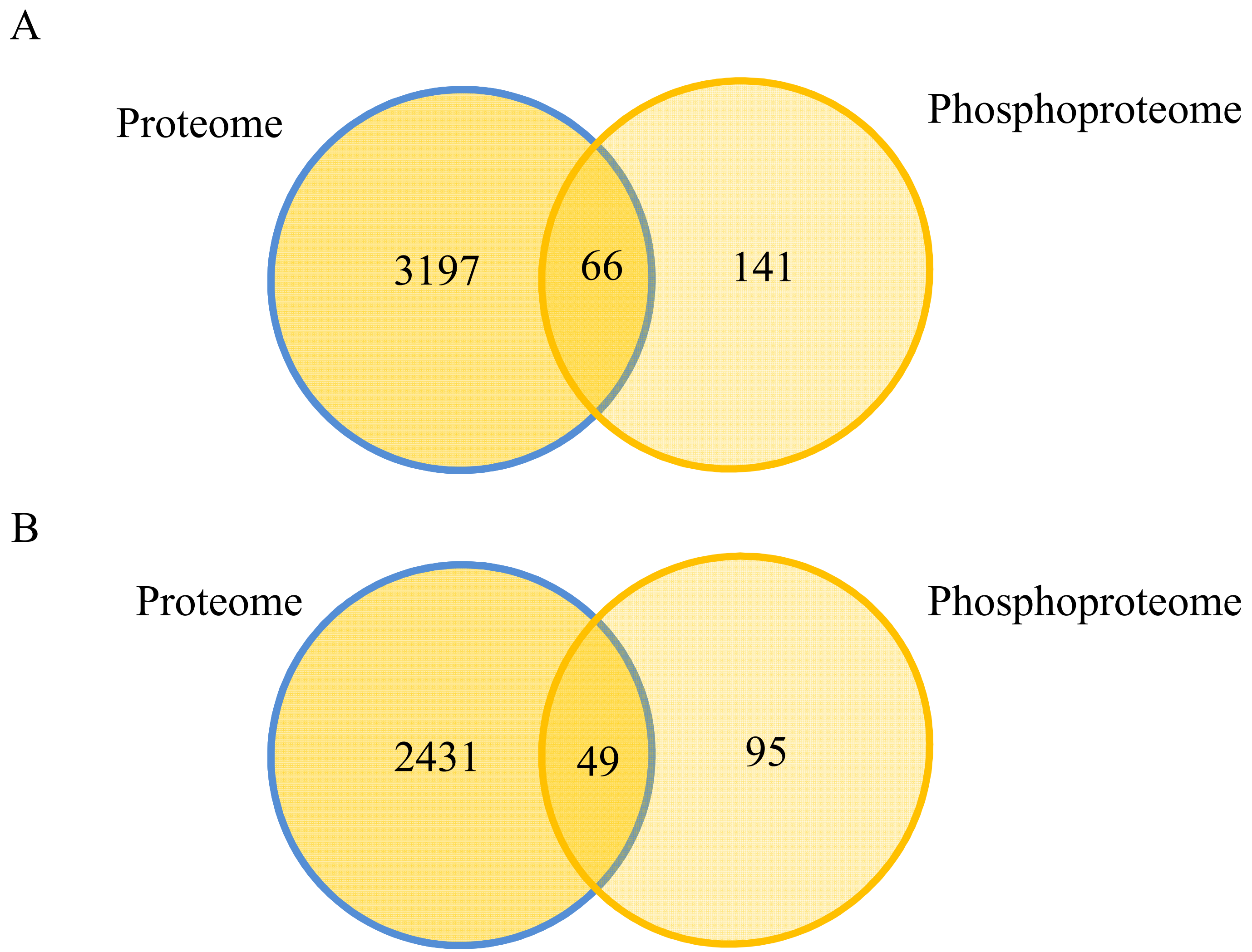

Supplementary Figure S4. Venn diagrams of number of proteins identified in Daojiao phosphoproteome and proteome. (A) The number of identified proteins; (B) The number of quantified proteins.

Supplemental Figure S5

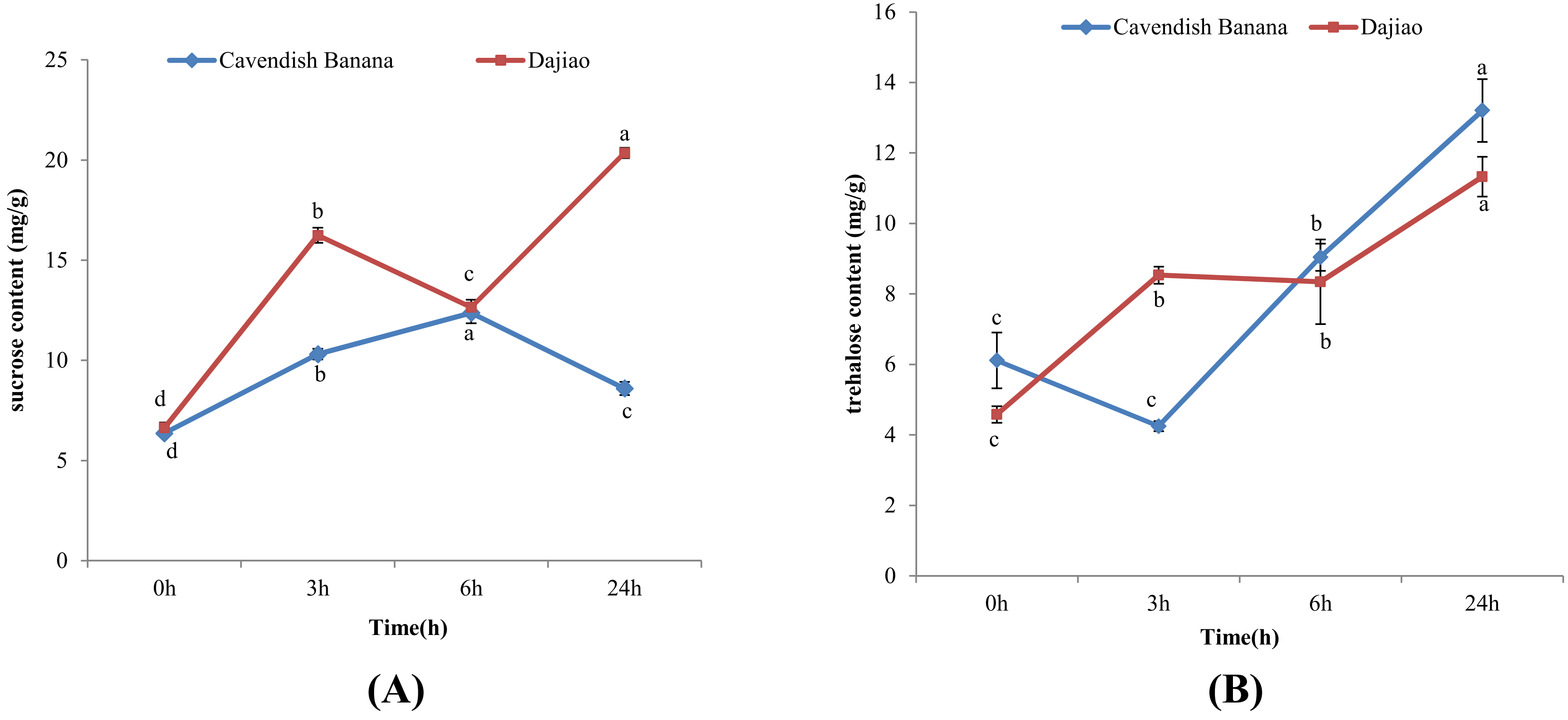

Supplementary Figure S5. (A) Sucrose content in Cavendish Banana and Dajiao under cold stress. (B) Trehalose content in Cavendish Banana and Dajiao under cold stress.

Table S2. Comparison of conserved phosphosites in Cavendish Banana, Dajiao with common plant species in UniprotKB database

Species	Phosphorylation [STY]			Un-phosphorylation [STY]			Fisher' exct test P value
	Conserved	unconseved	Conserved percent	Conserved	unconserved	Conserved percent	
Vitis vinifera	157	601	20.71%	7347	33224	18.11%	0.01994
Oryza sativa	140	618	18.47%	6393	34178	15.76%	0.01343
Arabidopsis thaliana	165	593	21.77%	8403	32168	20.71%	0.17187
Glycine max	154	604	20.32%	6507	34064	16.04%	0.00043
Sorghum bicolor	165	593	21.77%	8088	32483	19.94%	0.06905
Solanum lycopersicum	170	588	22.43%	7666	32905	18.90%	0.00379
Brachypodium distachyon	168	590	22.16%	8333	32238	20.54%	0.09116
Zea mays	123	635	16.23%	5787	34784	14.26%	0.04398
**Average result**			**20.48%**			**18.03%**	

Table S4. The information of 6 MEKK1 proteins, 2 MAPK4 proteins and 1 MAPK6

Protein accession	Protein names
GSMUA_Achr10P02140_001	Putative Mitogen-activated protein kinase kinase kinase 1
GSMUA_Achr10P25540_001	Mitogen-activated protein kinase kinase kinase 1
GSMUA_Achr11P13500_001	Mitogen-activated protein kinase kinase kinase 1
GSMUA_Achr1P12210_001	Putative Mitogen-activated protein kinase kinase kinase 1
GSMUA_Achr2P07690_001	Mitogen-activated protein kinase kinase kinase 1
GSMUA_Achr7P11850_001	Putative Mitogen-activated protein kinase kinase kinase 1
GSMUA_Achr4P03760_001	Mitogen-activated protein kinase 4
GSMUA_Achr4P11270_001	Mitogen-activated protein kinase 4
GSMUA_Achr11P02860_001	Mitogen-activated protein kinase 6
